# The role of cellular immunity in Influenza H1N1 population dynamics

**DOI:** 10.1186/1471-2334-12-329

**Published:** 2012-11-28

**Authors:** Venkata R Duvvuri, Jane M Heffernan, Seyed M Moghadas, Bhargavi Duvvuri, Hongbin Guo, David N Fisman, Jianhong Wu, Gillian E Wu

**Affiliations:** 1 Centre for Disease Modelling, York University, Toronto, Ontario; 2 Department of Biology, York University, Toronto, Ontario; 3 Department of Mathematics & Statistics, York University, Toronto, Ontario; 4 School of Kinesiology and Health Sciences, York University, Toronto, Ontario; 5 Dalla Lana School of Public Health, University of Toronto, Toronto, Ontario

## Abstract

**Background:**

Pre-existing cellular immunity has been recognized as one of the key factors in determining the outcome of influenza infection by reducing the likelihood of clinical disease and mitigates illness. Whether, and to what extent, the effect of this self-protective mechanism can be captured in the population dynamics of an influenza epidemic has not been addressed.

**Methods:**

We applied previous findings regarding T-cell cross-reactivity between the 2009 pandemic H1N1 strain and seasonal H1N1 strains to investigate the possible changes in the magnitude and peak time of the epidemic. Continuous Monte-Carlo Markov Chain (MCMC) model was employed to simulate the role of pre-existing immunity on the dynamical behavior of epidemic peak.

**Results:**

From the MCMC model simulations, we observed that, as the size of subpopulation with partially effective pre-existing immunity increases, the mean magnitude of the epidemic peak decreases, while the mean time to reach the peak increases. However, the corresponding ranges of these variations are relatively small.

**Conclusions:**

Our study concludes that the effective role of pre-existing immunity in alleviating disease outcomes (e.g., hospitalization) of novel influenza virus remains largely undetectable in population dynamics of an epidemic. The model outcome suggests that rapid clinical investigations on T-cell assays remain crucial for determining the protection level conferred by pre-existing cellular responses in the face of an emerging influenza virus.

## Background

In spring 2009, a new triple reassortant strain of the influenza A/H1N1 virus emerged. Exhibiting its unique genome composition with rapid global spread [1], this new strain caused the first pandemic of the 21st century. The genome combination of the 2009 H1N1 strain was antigenically distinct from the circulating seasonal influenza subtypes, seasonal H1N1 (sH1N1) and H3N2 [2,3]. With the exception of older individuals (> 60 years of age), all other age-groups did not confer any effective cross-antibody protection against this strain [4,5]. The lack of cross-reactive neutralizing antibodies in a large fraction of the population made the global spread of the virus readily possible. However, compared to the severity of past pandemics of 1918 H1N1, 1957 H2N2 and 1968 H3N2 [6], the 2009 H1N1 pandemic appeared relatively mild with a death toll of ≈ 300,000 resulting from respiratory and cardiovascular complications globally [7]. The unexpected mild nature and observed prolonged incubation period for this strain [8-10], received an increased attention towards the role of pre-existing cellular immunity. Population-wide studies suggest the existence of an immune response induced from conserved epitopes between the 2009 H1N1 and seasonal influenza strains [11-13]. An immunoinformatics study on the conservancy analysis and influenza epitope prediction [11] revealed the high level existence of Hemagglutinin (HA) CD4^+^ T cell epitope conservancy (≈ 95*%*−100*%*) between the sH1N1 and 2009 H1N1 isolates. However, no considerable conservancy between subtypes H3N2 and 2009 H1N1 was observed. This investigation hypothesized that the availability of cross-conserved epitopes and Major Histocompatibility Complex class II, HLA-DRB1 alleles essential to the activation of the T cell repertoire, may have played a role in reducing the severity of the 2009 H1N1 infection despite the lack of virus-specific antibody titres. A recent human experimental study demonstrated that conserved and common epitope-specific pre-existing CD4^+^ T cell immunity, but not CD8^+^, plays a critical role in limiting viral shedding and severity of infection in the absence of antibody titers [13]. Other studies have also shown recall responses of CD4^+^T helper memory cells to these shared conserved epitopes [14,15].

In this paper, we developed a mathematical model to explore the interplay between the individual cellular cross-reactivity and the population spread of disease, linking micro-dynamics with macro-dynamics within an immuno-epidemiology framework. The goal is to determine whether the cross-reactivity reported in our previous work [11] can be observed at the population level infection dynamics, i.e., to measure the relative change in the magnitude and timing of the epidemic peak.

## Methods

Our methodology is based on the development of a continuous time Monte-Carlo Markov Chain (MCMC) model to simulate the transmission dynamics of a novel influenza virus by taking into account pre-existing immunity as a result of prior exposure to seasonal influenza strains.

### Model

**Figure 1:**
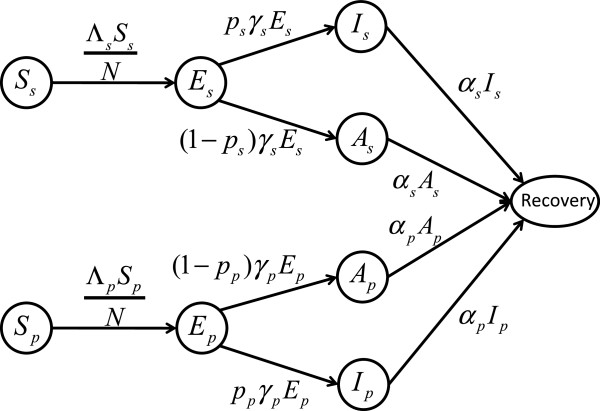
**Model diagram.** Dynamics of infection and movements of individuals between subpopulations.

We divided the population into classes of individuals susceptible to the new virus with no prior exposure (*S*_*s*_) and with prior exposure (*S*_*p*_) having some level of pre-existing T-cell immunity. Upon exposure to the virus, these individuals move to the exposed classes (*E*_*s*_, *E*_*p*_), and after the exposed period has elapsed move to the asymptomatically infected classes (*A*_*s*_, *A*_*p*_) or symptomatically infected classes (*I*_*s*_, *I*_*p*_). Finally, infected individuals move to the recovered class (*R*) upon recovery, gaining immunity against re-infection. A schematic of our model is provided in Figure 1. We defined ⋀_*s*_ and ⋀_*p*_ as the force of infection for the *S*_*s *_and *S*_*p *_populations, given by

(1) $$\begin{array}{c} {⋀}_{s}=\beta (I_{s}+I_{p}+\delta_{a}(A_{s}+A_{p})) \end{array}$$

(2) $$\begin{array}{c} {⋀}_{p}=\delta_{p}{⋀}_{s} \end{array}$$

where *β* is the baseline transmission rate, 0 <* δ*_*a *_< 1 denotes a reduction in transmissibility of asymptomatic infection, and 0 <* δ*_*p *_< 1 represents a possible reduction in the susceptibility of individuals in the *S*_*p *_subpopulation. We note that in the absence of specific antibodies, the reduction in susceptibility to infection will likely be very low (*δ*_*p *_≈ 1), even in the presence of T-cell immunity. However, this cellular immunity can reduce infectiousness (i.e., that corresponds to a reduction in transmissibility in the model), and mitigate illness (i.e., which corresponds to a higher probability of undergoing asymptomatic infection) [13].

The stochastic model for the transmission dynamics of infection is described in Table 1. The MCMC simulation moves forward in time through the determination of transition probabilities which, in turn, determine the amount of time that elapses between events. We obtained transition probabilities in the MCMC simulation using the rates defined in Table 1. These transition probabilities were then compared to random numbers generated in the simulation and this determines which event will occur. The classes of subpopulations were subsequently adjusted to reflect this event and new transition probabilities were obtained. This procedure continued until infection was wiped out from the population (i.e., when the exposed and infected classes were equal to zero).

**Table 1:** Stochastic dynamics of the model

**Event**	**Transition**	**Transition**
	**during Δ*t***	**rate**
Infection of a	*S*_*s*_→*S*_*s*−1_	$\frac{{⋀}_{s}S_{s}}{N}\Delta t+o(\Delta t)$
susceptible in *S*_*s*_		
Infection of a	*S*_*p*_→*S*_*p*_−1	$\frac{{⋀}_{s}S_{p}}{N}\Delta t+o(\Delta t)$
susceptible in *S*_*p*_		
Increase in exposed	*E*_*s*_→*E*_*s*_ + 1	$\frac{{⋀}_{s}S_{s}}{N}\Delta t+o(\Delta t)$
class *E*_*s*_		
Increase in exposed	*E*_*p*_→*E*_*p*_ + 1	$\frac{{⋀}_{s}S_{p}}{N}\Delta t+o(\Delta t)$
class *E*_*p*_		
Decrease in exposed	*E*_*s*_→*E*_*s*_−1	*γ*_*s*_*E*_*s*_*Δt* + *o*(*Δt*)
class *E*_*s*_		
Decrease in exposed	*E*_*p*_→*E*_*p*_−1	*γ*_*s*_*E*_*p*_*Δt* + *o*(*Δt*)
class *E*_*p*_		
Increase in asymptomatic	*A*_*s*_→*A*_*s*_ + 1	(1−*p*_*s*_)*γ*_*s*_*E*_*s*_*Δt* + *o*(*Δt*)
infection *A*_*s*_		
Increase in asymptomatic	*A*_*p*_→*A*_*p*_ + 1	(1−*p*_*p*_)*γ*_*p*_*E*_*p*_*Δt* + *o*(*Δt*)
infection *A*_*p*_		
Recovery from asymptomatic	*A*_*s*_→*A*_*s*_−1	*α*_*s*_*A*_*s*_*Δt* + *o*(*Δt*)
infection *A*_*s*_		
Recovery from asymptomatic	*A*_*p*_→*A*_*p*_−1	*α*_*p*_*A*_*p*_*Δt* + *o*(*Δt*)
infection *A*_*p*_		
Increase in symptomatic	*I*_*s*_→*I*_*s*_ + 1	*p*_*s*_*γ*_*s*_*E*_*s*_*Δt* + *o*(*Δt*)
infection *I*_*s*_		
Increase in symptomatic	*I*_*p*_→*I*_*p*_ + 1	*p*_*p*_*γ*_*p*_*E*_*p*_*Δt* + *o*(*Δt*)
infection *I*_*p*_		
Recovery from symptomatic	*I*_*s*_→*I*_*s*−1_	*α*_*s*_*I*_*s*_*Δt* + *o*(*Δt*)
infection *I*_*s*_		
Recovery from symptomatic		
infection *I*_*p*_		

Transition events during a *Δt*period of time.

### Parameterization of the model

We considered a population of 2000 susceptible individuals, and divided it into two groups based on their infection history with seasonal influenza A virus strains, H3N2 and sH1N1. Data from 2001 to 2009 indicate that subtype H3N2 has been the dominant circulating strain, making up over 80% of the viruses typed and subtyped in Europe [16,17]. Thus, we assumed an upper bound of 20% of individuals considered susceptible to the new virus with prior exposure to the sH1N1 strain in the pre-pandemic era, and considered a lower bound of 80% of the population fully susceptible with no prior exposure to sH1N1.

Cross-reactive pre-existing T-cell memory responses can provide protection by reducing the susceptibility of individuals *S*_*p*_ (this may be very low reduction), and decreasing the severity of disease and its transmissibility [13,18-20]. We assumed that the 2009 H1N1 susceptibility of individuals with prior exposure to sH1N1 is reduced due to T-cell cross-reactivity of conserved epitopes (by a factor *δ*_*p*_), but remains unchanged for those who had no prior exposure to sH1N1 (see [11,12]). Cross-reactivity was assigned at a level of 52% for *S*_*p*_, based on conserved T-cell epitopes [11,12]. To reflect the reduction in the severity of illness and transmission, we first assumed that the probability of an exposed individual in *E*_*p*_developing symptomatic infection was reduced compared to that of an exposed individual in *E*_*s*_, i.e., 0 <* p*_*p *_<* p*_*s *_< 1. We also assumed that the transmission of infection from asymptomatically infectious individuals is reduced (by a factor *δ*_*a*_) compared to symptomatically infectious cases. In our model, we let the latent period 1/*γ*_*p*_(for those with prior exposure to sH1N1) vary between the length of the latent period 1/*γ*_*s*_ and the total period of infection 1/*γ*_*s*_ + 1/*α*_*s*_(for those with no prior exposure to sH1N1). Furthermore, it was assumed that the total illness period following exposure is the same for all infected individuals (i.e., symptomatic and/or asymptomatic). Summarizing the above assumptions, we may highlight two main points: individuals with prior exposure to sH1N1 have (i) lower infectiousness and therefore lower transmissibility if infectious; and (ii) lower probability of developing symptomatic infection if exposed; compared to those with no prior exposure.

Note that existing literature does not provide any information on the total period of infection (i.e., 1/*γ* + 1/*α*) following exposure. While a prolonged incubation period has been reported as a result of pre-existing immunity [10], clinical experiments suggest a reduced period of illness [13]. Due to the lack of sufficient data and information, we have steered clear of changing the period of infection for those with prior exposure, and assumed that 1/*γ*_*p*_ + 1/*α*_*p *_= 1/*γ*_*s*_ + 1/*α*_*s*_. Parameter values for our model described in Table 2 are derived from the above assumptions and the published literature for seasonal and the 2009 H1N1 pandemic. We seeded simulations with initial numbers of infections *I*_*s*_(0) = 4 and *I*_*p*_(0) = 2. These initial values were chosen so that infection would progress in the population (i.e., if these were too small many simulations would clear the infection due to stochasticity).

**Table 2:** Model parameters

**Parameter**	**Description**	**Baseline values**	**Reference**
*R*_0_	Basic reproduction number	1.4 (range: 1.25−1.8)	[10,21-24]
*δ*_*p*_	Reduction in susceptibility of *S*_*p*_	0−1 (0.48 in simulations)	[11]
*δ*_*a*_	Reduction in transmissibility of *A*_*s*_ and *A*_*p*_	0.5	[25]
*p*_*s*_	Fraction of *E*_*s*_ that become symptomatic	0.6	[25,26]
*p*_*p*_	Fraction of *E*_*p*_ that become symptomatic	0.3	Assumption
1/*γ*_*s*_	Exposed period of *E*_*s*_	1.5 (days)^−1^	[27]
1/*γ*_*p*_	Exposed period of *E*_*p*_	1.5, 2.6, 4.3, 6 (days)^−1^	[10]
1/*α*_*s*_	Infectious period of *I*_*s*_ and *A*_*s*_	5 (days)^−1^	[25,26,28,29]
1/*α*_*p*_	Infectious period of *I*_*p*_ and *A*_*p*_	varied in simulations	[10]
*β*	Baseline transmission rate	variable	Estimated from *R*_0_
			expression in (3)

Description of parameters with their values and ranges extracted from the published literature.

### Basic reproduction number

The basic reproduction number (denoted by *R*_0_) is defined as the the average number of secondary cases generated by a single infectious case introduced into an entirely susceptible population [30,31]. According to this definition, it is expected that an epidemic will occur if *R*_0_ > 1, and die out if *R*_0_ < 1. For our model, *R*_0_ was determined to be:

(3) $$R_{0}=\left(\right)\frac{p_{s}+(1-p_{s})\delta_{a}}{\alpha_{s}}))\frac{\beta S_{s}^{0}}{N_{0}}+\left(\right)\frac{p_{p}+(1-p_{p})\delta_{a}}{\alpha_{p}}))\frac{\delta_{p}\beta S_{p}^{0}}{N_{0}}$$

where *N*_0_, $S_{s}^{0}$, $S_{p}^{0}$ correspond to the initial sizes of the total population, the population of susceptibles with no cross-reactivity, and the population of susceptibles with 52% cross reactivity (considered as the level of immunity), respectively. The reader may consult [30] for a review of different methods of *R*_0_ derivation. Other parameters in the expression of *R*_0_are described in the model diagram illustrated in Figure 1. We used estimates of *R*_0_ to calculate the transmission rate *β* for a given set of parameter values from Table 2. Reported *R*_0_ values for initial spread of the 2009 H1N1 virus lie in the range 1.4−1.6 in Mexico, 1.7−1.8 in the United States, and 1.25−1.38 in Ontario, Canada [10,21-24]. Note that, *R*_0_ from these studies is, in fact, a measurement of the effective reproduction number (denoted by *R*_*e*_) since prior immunity exists in the initial population [32]. To be consistent with [10,21-24], we refer to these measurements as *R*_0_.

## Results and discussion

**Figure 2:**
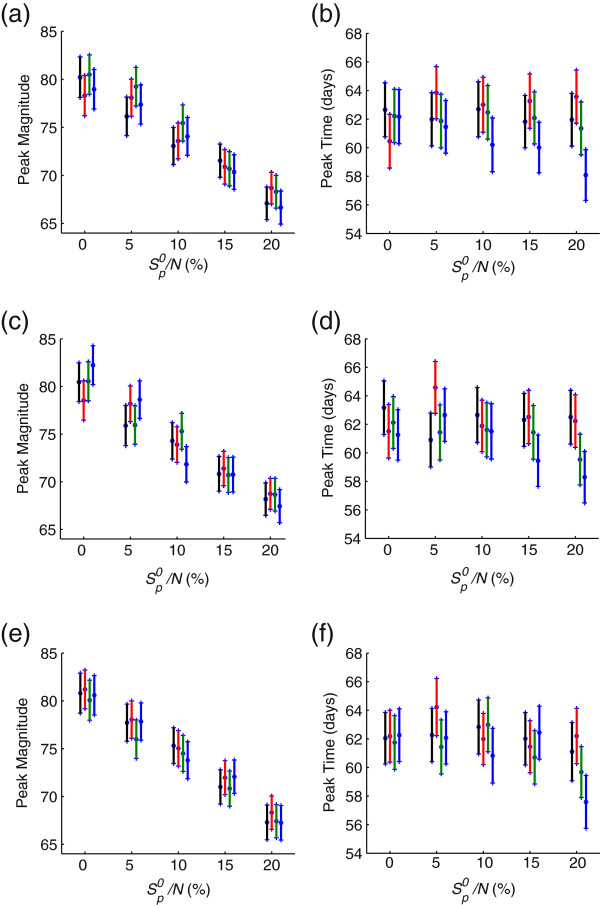
**Model simulations.** Variations in peak magnitude and peak time of the infection curve for the *A*_*s*_ + *I*_*s*_subpopulation. The mean (star) and standard deviation (bars) correspond to 1000 simulation runs for the initial population with cross-reactivity ( $S_{p}^{0}/N$) of 5%, 10%, 15%, and 20%, and an infectious period of 5 days (black), 3.9 days (red), 2.2 days (green), and 0.5 days (blue). The fraction of infected individuals (with prior exposure to sH1N1) which develops symptomatic infection is: (**a**,**b**) *p*_*p *_= 0; (**c**,**d**) *p*_*p *_= 0.5*p*_*s*_; and (**e**,**f**) *p*_*p *_=* p*_*s*_.

Since immune cross-reactivity is expected to prolong the incubation period and reduce the severity of illness [13,18-20], we simulated the stochastic dynamical model to investigate the effect of these factors in the epidemic profile. Simulations were run and mean and standard deviation (for 1000 runs) were recorded, when the initial population with cross-protection varies from 5% to 20% of the total population, the infectious period decreases from 5 to 0.5 days, and the proportion of the exposed class with cross protection *E*_*p*_ becoming symptomatic *I*_*p*_ varies from 0% to 60%. Figure 2 shows the variation in the peak magnitude (left column) and time to this peak (right column) for the infectious subpopulation *I*_*s*_ + *A*_*s *_when *p*_*p *_= 0 (top), 0.05*p*_*s*_ (middle) and *p*_*s*_(bottom). In each scenario, the exposed period lasts for on average 1.5 (black), 2.6 (red), 4.3 (green), 6 (blue) days, and the initial size of the population with cross protection $S_{p}^{0}$ varies from 5% to 20%. The figure shows the mean of the simulation runs and two standard errors from the mean, where (standard error = SD/(number of simulations)). These simulations demonstrate that a change in the peak magnitude (Figure 2, left column) can result from a change in the initial size of the population with pre-existing immunity, but the change in infectious period and the fraction of *E*_*p*_ moving to the *I*_*p *_class have virtually no effect. The time of the peak magnitude was not affected by any change in these model parameters (Figure 2, right column).

To determine the effect of cross protection *δ*_*p *_on the peak magnitude and time to peak, we also determined these data points for the case where cross reactivity provided no protection to individuals previously exposed to sH1N1 (*δ*_*p *_= 1), and only provided reduced illness and transmission. Again, we observed that, as the initial size of the population with prior exposure to sH1N1 increases, the peak magnitude of *A*_*s*_ + *I*_*s *_subpopulation decreases, but the time to peak was not affected. We also observed that variation in the exposed period from 1.5−6 days and the fraction of the *E*_*p*_ class moving to the infectious symptomatic class *I*_*p *_from 0 <* p*_*p *_<* p*_*s *_played no significant effect in the model outcomes. The results from these simulations differed very little from the case when cross protection was included in the simulations (when *δ*_*p *_= 0.48; simulations not shown). Further simulations demonstrate that our results hold true for larger population sizes of susceptible individuals.

During an epidemic, infected individuals who can be identified (either as influenza-like illness or laboratory confirmed case) constitute a fraction of the subpopulation *I*_*s*_ with symptoms. In our simulations, the peak magnitude of this subpopulation changes very little, and the time to peak is unchanged from that found above. Hence it is unlikely that the impact of pre-existing cellular immunity would be observed in passive surveillance data associated with case reporting.

## Conclusion

The 2009 H1N1 pandemic has been characterized as the mildest pandemic on record [8-10]. Typically, disease was mild in all age groups, which is not common to all pandemics [8]. Recent studies of the 2009 H1N1 pandemic suggest that this may be attributed to the host counter responses to infection, induced from the pre-existing CD4^+^ and CD8^+^T cell responses to the conserved epitopes [18-20]. Our previous work found that there is a high CD4^+^ T-cell epitope conservancy and Major Histocompatibility Complex (MHC) class II, HLA-DRB1 promiscuity between sH1N1 (which has been circulating for some 33 years), and the 2009 H1N1 pandemic strain [11]. We hypothesized that the consistent circulation and infective ability of the sH1N1 strain may be a critical factor to the atypical mild nature of 2009 H1N1, because both strains shared common and conserved epitopes, which remain intact in evolutionary mechanisms. In the current work, we extended this epitope conservancy study to explore the interplay between individual cross-reactivity and the population dynamics of disease spread using the MCMC simulation approach.

We developed a simulation model to determine the effect of cross-reactivity on the dynamics of H1N1 infection in a population. The results were used to compare infection curves in the presence and absence of cross-reactivity, and when the initial size of the population with pre-existing immunity to the new strain (resulted from previous exposure to sH1N1) changes. We found that the infection curves of the asymptomatically and symptomatically infected populations undergo a very small change in peak magnitude and peak time. While pre-existing immunity can reduce the likelihood of clinical disease and mitigate illness, our findings suggest that the effects of T-cell immunity would be unlikely to be observed through only surveillance data collected for influenza-like illnesses or laboratory confirmed cases. This has important implications for public health planning and the development of targeted strategies (e.g., vaccination) for mitigating the impact of disease in the population. First, the incidence of an influenza epidemic may not provide a reliable measurement on the novelty of the virus or the level of pre-existing immunity in the population. Second, the effect of pre-existing cellular immunity in alleviating disease outcomes (e.g., hospitalization) remains largely undetectable in population dynamics of an epidemic. These suggest that rapid clinical investigations on T-cell assays remain crucial for determining the protection level conferred by pre-existing cellular responses in the face of an emerging influenza virus.

Our study focused on a fixed measurement of *R*_0_, varying the transmission rate (*β*) and the initial proportion of the population ( $S_{p}^{0}$). A similar interesting question related to our current study is to determine how peak magnitude and time change if *β* is fixed and $S_{p}^{0}$ is varied, which will change *R*_0_[32]. This is a course for future work.

Our study focused on the effect of T-cell immunity developed from sH1N1 in providing partial protection against infection caused by a novel influenza strain. In the context of the 2009 H1N1 pandemic, previous work in primates has shown sH1N1 primed animals cleared the infection rapidly, suggesting the role of cross-reactive T-cell responses [33]. In the absence of specific protective antibodies, epitope-specific CD4^+^ T cell immunity can help reduce viral shedding and mitigate illness, with evidence of cytolytic activity [13]. In future work, combining the effects of both (cellular and humoral) types of immunity will be an important step in determining how immune system dynamics will impact the epidemic course, which may provide critical information that can be used to project plausible patterns of disease spread in the population at the early stages of disease outset.

## Competing interests

DF has received past research and educational funding from Sanofi Pasteur, Glaxo Smith Kline, and Novartis, all of which manufacture vaccines against influenza.

## Author’s contributions

VD, SMM, JMH and DF were involved in the development of the model. VD, HG and JMH carried out the simulations. VD, JMH and SMM carried out the model analysis. BD participated in model analysis. VD, JMH, SMM wrote the mansucript. DF, BD, HG, JW and GW editted the mansucript. All authors read and approved the final manuscript.

## Pre-publication history

The pre-publication history for this paper can be accessed here:

http://www.biomedcentral.com/1471-2334/12/329/prepub
